# Chemical Characterization, Analgesic, Antioxidant, and Anticholinesterase Potentials of Essential Oils From *Isodon rugosus* Wall. ex. Benth

**DOI:** 10.3389/fphar.2018.00623

**Published:** 2018-06-13

**Authors:** Abdul Sadiq, Anwar Zeb, Farhat Ullah, Sajjad Ahmad, Muhammad Ayaz, Umer Rashid, Noor Muhammad

**Affiliations:** ^1^Department of Pharmacy, University of Malakand, Chakdara, Pakistan; ^2^Department of Chemistry, COMSATS University Islamabad, Abbottabad, Pakistan; ^3^Department of Biotechnology and Genetic Engineering, Kohat University of Science and Technology, Kohat, Pakistan

**Keywords:** essential oil, GC-MS, *Isodon rugosus*, antinociception, opioid receptors, antioxidant, anticholinesterase

## Abstract

*Isodon rugosus* Wall. ex. Benth is an important species and is used in folk medicine for different types of pains such as abdominal pain, earache, toothache, gastric, and generalized body pain. Recently, we also have reported the antinociceptive potential of chloroform fraction of *I. rugosus*. In this research, we have investigated the antinociceptive, antioxidant and anti-cholinesterase potentials of essential oils from *I. rugosus* (Ir.EO), and have determined a possible mechanism of anti-nociception. The Ir.EO was subjected to gas chromatography-mass spectroscopy analysis to find out its chemical constituents. The Ir.EO was assayed for analgesic potential following acetic acid induced writhing, formalin test and hot plate method in animal models. The antioxidant activity was conducted against DPPH and ABTS free radicals following spectroscopic analysis. The cholinesterase inhibitory assays were performed using Ellman's assay. The GC-MS analysis of Ir.EO revealed the identification of 141 compounds. Ir.EO demonstrated strong antinociceptive potential in all three *in-vivo* models. With the use of nalaxone, it was confirmed that the essential oil was acting on the central pathway of nociception. The Ir.EO also exhibited strong free radicals scavenging potential, exhibiting IC_50_ values of 338 and 118 μg/ml for DPPH and ABTS free radicals respectively. In AChE and BChE inhibitory assays, the observed IC_50_ values were 93.56 and 284.19 μg/ml respectively. The encouraging antinociceptive, antioxidant and anticholinesterase results revealed that Ir.EO is a rich source of bioactive compounds as obvious from the GC-MS results.

## Introduction

Globally, a large number of medicines are available for the treatment of pain and associated disorders. Non-steroidal anti-inflammatory drugs (NSAIDs) are widely used for the management of pain and inflammation due to their strong efficacy (Zarin et al., 2005). However, their use is associated with severe side effects. Alternatively, the drugs from natural origins are considered to be relatively safe and are associated with fewer unwanted effects. Natural products, especially the plants play a vital role in the discovery of new chemical entities with potential therapeutic values (Rates, 2001; Ayaz et al., 2017a). The traditional use of plants is therefore a logical strategy to find out natural therapeutic agents for different ailments like pain and inflammation (Gupta et al., 2006). Despite the development of therapeutic agents for pain, there is still a demand to search out novel agents which could treat pain and related disorders more efficiently (Calixto et al., 2000).

The reactive oxygen species (ROS) are produced within the body as a result of redox processes and aerobic respiration. These ROS invade lipids, proteins, enzymes, DNA and RNA and ultimately damage the cells. These biomolecules play a vital role in stimulation, propagation and maintenance of inflammatory processes, as well as pain and neurodegenerative disorders (Zhu et al., 2004). These unwanted effects can be reduced by the use of antioxidants which either reduce the production of ROS or diminish them before reaction (Khalil et al., 1999; Cuzzocrea et al., 2001). In this regard, the essential oils isolated from herbal sources may be considered for the management of pain, inflammation, and free radicals scavenging. Several plants have been reported with strong antioxidant potentials against free radicals (Ahmad et al., 2015; Ayaz et al., 2015).

Alzheimer's disease (AD) is a common neurodegenerative disorder characterized by cognitive hypo-function, behavioral turbulence and difficulties in life activities (Ali et al., 2017; Ayaz et al., 2017b). AD is believed to be the major cause of dementia in elder population (Ullah et al., 2016). According to the statistics, 27 million people are affecting globally from Alzheimer and is a major life threat after cancer and cardiovascular diseases (Hebert et al., 2003). AD pathogenesis include synaptic deficiency of essential neurotransmitter (acetylcholine, ACh) which is implicated in the neurotransmission (Sadiq et al., 2015). Other aspects of AD include accumulation of amyloid beta (Aβ), neurofibrillary tangles (NFTs), and free radicals induced neurodegeneration (McLean et al., 1999; Zeb et al., 2014a; Ahmad et al., 2016). The inhibition of cholinesterase is a vital biochemical target involved in the degradation of ACh which increases its accumulation in the synaptic region. Among the five clinically approved anti-Alzheimer drugs, four are cholinesterase inhibitors while the fifth drug memantine is glutametergic system modifier. Despite the fact that several anti-amyloid and anti-NTFs drugs are in clinical trials, but, till date, no one is approved for clinical use. Furthermore, administration of free radical scavengers is also an important strategy, as Aβ is potent generator of free radicals and a mitochondrial poison. Plants are a source of mutli-potent drugs, including anti-AD drugs. Among the currently available anti-AD drugs, physostigmine, and galanthamine are derived from medicinal plants (Ahmad et al., 2016). Furthermore, natural products are free radicals' scavengers and can be effective on multiple pathways (Ayaz et al., 2017c).

*Isodon rugosus* Wall. ex. Benth. is a well-known species of family Labiateae. The bark of *I. rugosus* is used ethnomedicinally in the treatment of dysentery and curing of body pain (Shuaib et al., 2015). Folklorically, the fresh leaves' extract of *I. rugosus* is applied to the effected skin and is also used for earache (Sabeen and Ahmad, 2009). Moreover, the dried leaves of this plant can be used for the treatment of teeth pain (Akhtar et al., 2013). The plant has also been reported to posses potential effectiveness in gastric and abdominal pains (Ahmad et al., 2014). Moreover, other traditional uses of *I. rugosus* are attributed to its possible use against infectious diseases, pyrexia, blood pressure, rheumatism, and in pain associated with teeth (Khan and Khatoon, 2007; Adnan et al., 2012; Shuaib et al., 2014). The extracts of *Isodon rugosus* have been previously published to posses certain biological potentials like anti-diarrheal, analgesic, antimicrobial, anticholinesterase, antioxidant, cytotoxic, phytotoxic, hypoglycemic, and as bronchodilator (Sher et al., 2011; Ajmal et al., 2012; Janbaz et al., 2014; Zeb et al., 2014a,b, 2016, 2017).

Based on the ethnomedicinal importance and our previously published work, this piece of research is designed to investigate the *in-vivo* analgesic mechanism, *in-vitro* antioxidant, and anti-cholinesterase activities of essential oils of *Isodon rugosus*.

## Methods

### Plant sample collection & isolation of essential oil

*Isodon rugosus* was collected from Dir (L), KP, Pakistan in July. The name *Isodon rugosus* was confirmed by Dr. Ali Hazrat, Department of Botany, Shaheed Benazir Bhutto University Dir (U), KP, Pakistan. The plant sample was stored for future record at the herbarium with voucher specimen number 1016AZ. The essential oils were extracted by hydrodistillation with the help of a Clevenger type apparatus (Lambert et al., 2001). The isolated essential oils were stored in refrigerator.

### Gas chromatography analysis

The phytocomponents of essential oils were separated using the same GC instrument as we previously reported (Ahmad et al., 2016). A capillary column having dimensions of 30 m × 0.25 mm with film thickness of 0.25 μm in combination with a flame ionization detector was used. The initial temperature was 70°C for 1 min, which was raised gradually to 180°C with 6°C/min increase for 5 min. Finally, the oven temperature was increased to 280°C with 5°C/min increase for 20 min. Temperature of the injector port was 220°C while that of detector was maintained at 290°C. Helium was used as a carrier gas. The sample was diluted in *n*-pentane (1/1,000, v/v) of 1 μl (Ayaz et al., 2016).

### GC-MS analysis

The GC-MS analysis of essential oil isolated from *Isodon rugosus* was determined with the previously reported parameters (Ayaz et al., 2015).

### Identification of components

The retention times and spectra of separated compounds by GC-MS were compared with the standard compounds for identifications. The mass spectrum of each separated compound with its fragmentation pattern was compared with the reported compounds (Stein et al., 2002; Adams, 2007).

### Experimental animals

The Swiss albino mice of either sex were used in analgesic experiments which were obtained from research laboratory of National Institute of Health, Islamabad, Pakistan. The animals were used as per the approval of the ethical committee, Department of Pharmacy, University of Malakand, Pakistan according to the animals Bye-Laws 2008 (Scientific Procedure Issue-1).

### Acute toxicity

Swiss albino mice were taken in various groups, having 5 test animals in each group. The essential oil samples were administered to the animals orally in different doses (250–2,000 mg/kg). To increase the aqueous solubility of essential oil, 0.1% v/v tween-80 (Sigma Aldrich)- was used. After administration of the doses, animals were critically observed for 72 h for hypersensitivity, abnormal behavior, and death. The experimental animals were observed for 20 days for sub-chronic effects and lethality (Hosseinzadeh et al., 2000).

### Analgesic activities

#### Acetic acid-induced writhing test

In acetic acid induced writhing test, the essential oil was administered orally (PO) in the same concentrations as mentioned in above section. After 30 min of interval, acetic acid (0.6%, 10 ml/kg) was injected into the mice intra-peritoneally. Tween-80 (0.5%, 3 ml/kg) was administered to Group I animals. The Group I was used as a negative control. The standard drug diclofenac sodium was administered to Group II with a dose of 10 mg/kg. The essential oil samples were administered to Groups III and IV in concentrations of 50 and 100 mg/kg respectively. After administration of acetic acid, the number of writhes were counted for 30 min (Franzotti et al., 2000).

#### Formalin test

The formalin-induced licking test of Ir.EO was carried out using Swiss albino mice weighing 25–30 gm. The test was performed in a controlled environmental temperature (23 ± 2°C) with light-dark cycle of 12 h each. Food and water was freely available to the test animals throughout the investigations. The essential oil was administered intraperitonially (I/P) to the experimental animals at various concentrations. After 30 min, 20 μl formalin (2.5%, v/v in distilled water) was injected subcutuneously (S/C) into the plantar surface of the hind paw. Tween-80 (0.5%, 3 ml/kg), a negative control in the experiment was administered to the Group I. Morphine (5 mg/kg), a standard drug, was administered to Group II animals. The animals in Groups III and IV were injected Ir.EO at concentrations of 50 and 100 mg/kg respectively. The nociceptive behavior was designated by formalin-induced licking of paw. The total time taken in the behavioral changes of the mice responses to nociception was recorded, such as licking and/or biting of the injected paw. The time taken was recorded for 30 min. The initial 5 min were considered as early phase, while 2nd period (15–30 min) as the late phase of the response. The early and late phase are termed as neurogenic and inflammatory phase, respectively (Sulaiman et al., 2008).

#### Hot plate test

The hot plate test method was assessed for the antinociception potential of essential oil isolated from *I. rugosus* as per the reported procedure (Zeb et al., 2016). In this method, a heated surface of a hot plate analgesia meter (Ugo Basile, model-7280) was maintained at 55 ± 0.2°C. The animals were kept over a heated surface in a closed glass cylinder. The time of the animals' placement and licking of hind paw or jumping over the heated surface were recorded as response latency. These are the parameters as a result of the thermal reactions. The oil samples, in concentrations of 50 and 100 mg/kg, while morphine 5 mg/kg, i.p., were administered 30 min before the beginning of the assessment. Mice were observed before administration of samples, and then at 30, 60 and 90 min after the samples taken. The cut-off time was 20 s.

#### Involvement of opioid receptors

This experiment was carried out to confirm the possible involvement of opioid receptors in the essential oil-induced antinociception. The procedure was evaluated using a hot plate and formalin test method as mentioned earlier. In this method, different groups of experimental mice (*n* = 6) were pretreated with naloxone (5 mg/kg, S/C), which is a non-selective opioid receptor antagonist. Naloxone was injected 15 min before the administration of Ir.EO and morphine.

### Antioxidant assays

#### DPPH assay

The DPPH free radicals scavenging effect was figured out for Ir.EO as previously published (Shah et al., 2015b). The DPPH solution (0.004%) in methanol was prepared which appeared with a deep violet color. Initially, the stock solution of essential oil with a known concentration of 1,000 μg/mL was prepared in ethanol. Then, this solution was diluted serially to obtain different concentrations from 62.5 to 1,000 μg/mL. Afterwards, 0.1 mL of the serially diluted concentration was added to 3.0 mL of DPPH solutions. This mixture was stored at dark place for 30 min at 23°C. After 30 min, the absorbance of each oil sample was measured by using double beam spectrophotometer at a wavelength of 517 nm. Ascorbic acid served as a positive control. The percent activity of all the samples was recorded as mean ± SEM. The percent radical scavenging potential was figured out using the following formula;

$$\begin{array}{l} \text{Scavenging effect \%}=\\ \;\frac{\text{control absorbance }-\text{ sample absorbance}}{\text{control absorbance}}\times 100 \end{array}$$

#### ABTS assay

Antioxidant potential of Ir.EO was also investigated using free radicals of 2, 2-azinobis [3-ethylbenzthiazoline]-6-sulfonic acid (ABTS) (Ullah et al., 2017). Solutions of ABTS (7 mM) and potassium persulfate (2.45 mM) were prepared and mixed thoroughly. The prepared solution was stored in a dark place overnight to generate free radicals. The absorbance of this solution was adjusted at 745 nm to 0.7 by addition methanol (50%). ABTS solution 3 mL was added to the test tubes containing samples having volume of 300 μL. The solution was transferred to the sample holder and absorbance was recorded for 6 min by using a double beam spectrophotometer. Ascorbic acid was used as a standard. The percent ABTS free radicals scavenging potential of the oil sample was measured by using the given formula;

$$\begin{array}{l} \text{Scavenging activity }\left(\text{\%}\right)\\ =\;\frac{\text{Absorbance of control }-\text{ Absorbance of Ir}.\text{EO}}{\text{Absorbance of control}}\;\times \;100 \end{array}$$

#### Anticholinesterase assays

Cholinesterases inhibitory potentials of Ir.EO was evaluated following Ellman's assay (Ellman et al., 1961). This procedure is based on enzymatic breakdown of substrates like acetylthiocholine iodide and butyrylthiocholine iodide by AChE and BChE respectively to form 5-thio-2-nitrobenzoate anions. The resultant anions consequently form a complex with DTNB and are converted into UV detectable yellow color compound. The formation of this compound is quantified in the presence and absence of inhibitor agents. In brief, 5 μL enzyme solution was added to each well of micro plate with subsequent addition of 5 μL DTNB solution. The resulting mixture was incubated for fifteen min at 30°C in water bath, and finally 5 μl substrate solution was added to it. At the end, absorbances were recorded at 412 nm. The control samples were the same as above mentioned but were without inhibitors. The change in absorbance was observed beside reaction time. The activity of enzymes and its inhibitory activities were determined for control as well as test samples from the rate of absorption with change in time as, V = Δ Abs /Δ t, and enzyme inhibition as;

$$\begin{array}{l} 100\;\times \;\frac{\text{V}}{{\text{V}}_{\max}} \end{array}$$

Where, V_max_ is enzyme activity in the absence of inhibitor agent.

#### Estimation of IC_50_ values

The median inhibitory concentration (IC_50_) values of DPPH, ABTS, AChE, and BChE inhibitory assays were find out by linear regression analysis of the percent inhibition versus concentrations of the test samples through MS Excel program (Shah et al., 2015a; Sadiq et al., 2016).

### Statistical data analysis

The values of all the tests were tabulated as mean ± S.E.M. Significant differences of the percent inhibitions of various test samples were analyzed via one way ANOVA following Bonferroni's post-test using GraphPad Prism software in which the *P* < 0.05 were considered significant.

## Results

### GC-MS analysis

The essential oil of *Isodon rugosus* was subjected to GC-MS analysis and total of 141 compounds were identified. On the given GC method, the retention times of the identified compounds were from 6.057 to 81.661 min. The details of all identified compounds are given in Table 1.

**Table 1:** List of all the compounds identified in the GC-MS analysis of essential oil of *Isodon rugosus*.

**S. No**.	**Compound label**	**RT**	**Name**	**Formula**	**Hits (DB)**
1	Methyl ethyl ketone	6.057	Butanone	C4H8O	10
2	2-cyclohexenyl vinyl ether	6.683	Na	C8H12O	10
3	Alpha.-Copaene	18.689	Alpha Copaene	C15H24	10
4	BETA-bOURBONENE	19.522	BETA. BOURBONENE	C15H24	10
5	8-Isopropyl-1-methyl-5-methylene-1,6-cyclodecadiene	19.605	Germacrene D	C15H24	10
6	2,6-Dimethylocta-1,4,7-triene	19.643	Cis-Achillene	C10H16	10
7	2-Butanone, 4-(2,2-dimethyl-6-methylenecyclohexyl)	19.764	Na	C13H22O	10
8	Bicyclo[3,3,1]non-2-ene, 7-oxa-2,8,9-trimethyl-5-acetoxymethyl	19.866	Na	C14H22O3	10
9	1,4-Dimethylpent-2-enyl)benzene	19.976	Na	C13H18	10
10	Beta.-Caryophyllen	20.312	Caryophyllene	C15H24	10
11	1,1,7-TRIMETHYL-4-METHYLENEDECAHYDRO-1H-CYCLOPROPA[E]AZULENE	20.584	AROMADENDRENE	C15H24	10
12	Alpha.-Cubebene	20.851	Alpha Cubebene	C15H24	10
13	CADINA-1,4-DIENE	20.937	Na	C15H24	10
14	3,8-Dimethylundecane	20.98	Na	C13H28	10
15	Cycloisolongifolene, 8,9-dehydro-	21.184	Na	C15H22	10
16	Epi-bicyclosesquiphellandrene	21.227	Na	C15H24	10
17	Alpha.-Amorphene	21.477	Alpha Amorphene	C15H24	10
18	Benzene, 1-(1,5-dimethyl-4-hexenyl)-4-methyl	21.549	Ar-Curcumene	C15H22	10
19	Cis-(-)-2,4a,5,6,9a-Hexahydro-3,5,5,9-tetramethyl(1H)benzocycloheptene	21.626	Na	C15H24	10
20	7-Methoxy-1,2,3,4-tetrahydro-9H-pyrido[3,4-b]indole	21.788	Na	C12H14N2O	6
21	Cadina-4,9-diene	21.977	Alpha.-Muurolene	C15H24	10
22	1-Hydroxy-1,7-dimethyl-4-isopropyl-2,7-cyclodecadiene	22.452	Germacrene D-4-ol	C15H26O	10
23	Calamenene	22.479	Calamenene	C15H22	10
24	9-Methyl-S-octahydrophenanathracene	22.603	Na	C15H20	10
25	2,5,9,9-Tetramethyl-6,7,8,9-tetrrahydro-5H-benzocycloheptane	22.775	Na	C15H22	10
26	5H-Inden-5-one, 1,2,3,3a,4,7a-hexahydro-7a-methyl-, trans-	22.823	Na	C10H14O	10
27	1,1,6-trimethyl-1,2-dihydro naphthalene	22.917	CALACORENE	C13H16	10
28	3-Heptadecen-5-yne	23.126	Na	C17H30	10
29	1,6,10-Dodecatrien-3-ol, 3,7,11-trimethyl	23.471	Trans-Nerolidol	C15H26O	10
30	6-Pentadecen-9-yne	23.885	Na	C15H26	10
31	Verbenene	23.923	Verbenene	C10H14	10
32	N-Butyl-3-hydroxybutyramide	24.061	Na	C8H17NO2	3
33	N-(1-Methylethyl)-2-(1-methylethyl)benzamide	24.073	Na	C13H19NO	10
34	Clov-2-ene-9.alpha.-ol	24.145	Na	C15H24O	10
35	VIRIDIFLOROL	24.221	Veridiflorol	C15H26O	10
36	Ledol	24.319	Ledol	C15H26O	10
37	1H-Inden-1-one, octahydro-, cis	24.462	Hydrindan	C9H14O	10
38	3-(Hydroxymethyl)-4-hydroxy-5,6,7,8-tetrahydroquinoline	24.531	Na	C10H13NO2	3
39	1,2-Naphthalenedione, 3,8-dimethyl-5-(1-methylethyl)	24.561	Mansonone C	C15H16O2	10
40	Humulane-1,6-dien-3-ol	24.618	Na	C15H26O	10
41	Cedr-8-ene	24.696	Alfa-cedrene	C15H24	10
42	Longifolenaldehyde	24.781	Longifolenaldehyde	C15H24O	10
43	2,3-Bis[(adamantylcarbonyl)ethynyl]bicyclo[2.2.1]hepta-2,5-diene	24.993	Na	C33H36O2	10
44	1,2-Diacetyl-4-methylbenzene	25.134	Na	C11H12O2	10
45	1.beta.,10.beta.H-Cadin-4-en-10-ol	25.392	T-Muurolol	C15H26O	3
46	4,4-Dimethylpentanenitrile	25.406	Na	C7H13N	1
47	Cadin-4-en-10-ol	25.57	Alpha.-Cadinol	C15H26O	10
48	5,7-Dimethylquinoline	25.583	Na	C11H11N	10
49	Azulene, 1,4-dimethyl-7-(1-methylethyl)	25.686	Azunol	C15H18	10
50	1,4-Methanobenzocyclodecene, 1,2,3,4,4a,5,8,9,12,12a-decahydro-	25.864	Na	C15H22	5
51	4,4a,5,6,7,8-Hexahydro-4a-methyl-2(3H)-naphthalenone	26.157	Na	C11H16O	10
52	7,7-dichlorobicyclo[3.2.0]hept-2-en-6-one	26.262	Na	C15H24O	10
53	6-Methylenebicyclo[2.2.1]hept2-en-1-ol	26.348	Na	C8H10O	1
54	MUUROLA-4,10(14)-DIEN-3-ONE	26.374	Na	C15H22O	10
55	Pentalene, octahydro-1-(2-octyldecyl)	26.513	Na	C26H50	10
56	1.beta.,4.beta.H,10.beta.H-Guaia-5,11-diene	26.627	Gamma.-Gurjunene	C15H24	10
57	Phosphorochloridic acid, diethyl ester	26.788	Diethyl chlorophosphate	C4H10ClO3	10
58	Isoisopulegyl acetate	26.902	Isoisopulegyl acetate	C12H20O2	10
59	1,3-dimethyl-3-acetoxymethyl-2-oxabicyclo[2.2.2]octan-5-one	27.314	Na	C12H18O4	10
60	Benzyl benzoate	27.873	Benzyl benzoate	C14H12O2	10
61	Ttrans-1-(3′′-Cyclopropylidenepropen-1′′-yl)-1-(propen-3′-yl)cyclopropane	28.23	Na	C12H16	5
62	Ethyl 3-phenylhexa-2,4-dienoate	29.066	Na	C14H16O2	4
63	1-(6′-Methoxy-7′-methyl-1′,2′,3′,4′-tetrahydronaphthalen-1′-yl)ethanol	29.312	Na	C14H20O2	2
64	6-(p-Tolyl)-2-methyl-2-heptenol	29.584	Nuciferol	C15H22O	10
65	2-Butanol, 4-[2,2,6-trimethylcyclohexyl]-, acetate	30.058	Tetrahydroionyl acetate	C15H28O2	10
66	1H-3a,7-Methanoazulene, octahydro-1,4,9,9-tetramethyl	30.327	Patchoulane	C15H26	10
67	Trans-Pinocarvyl acetate	30.688	Na	C12H18O2	3
68	[1-(R,S),5(R,S)]-3-(2,2-dimethyl-1-(R,S/S,R)-hydroxypropyl)-6-(R,S)-n-octyl-…	31.48	Na	C18H32O3	4
69	3-Angelate of felikiol	31.704	Na	C20H32O4	1
70	2-thia-6-methyl-7-(2-formylethyl)bicyclo[3.2.0]hept-6-ene-2,2-dioxide	31.795	Na	C10H14O3S	3
71	Aphanamol	32.079	Aphanamol	C15H24O2	10
72	5,9-Undecadien-2-one, 6,10-dimethyl	32.317	Geranylacetone	C13H22O	10
73	4-Chlorobutyric acid, octadecyl ester	32.639	Na	C22H43ClO2	10
74	Bicyclo[5.2.0]nonane, 4-methylene-2,8,8-trimethyl-2-vinyl-	32.976	Na	C15H24	10
75	2,9-Dimethyl-8-oxatetracyclo[5.4.1.1(3,10).0(5,9)]tridecane-2-endo,7-diol	33.499	Na	C14H22O3	10
76	1-(4-Hydroxy-3-isopropenyl-4,7,7-trimethyl-cyclohept-1-enyl)-ethanone	33.918	Na	C15H24O2	7
77	1,2-pentanediol, 5-(6-bromodecahydro-2-hydroxy-2,5,5a,8a-tetramethyl-1-napht…	34.228	Na	C20H35BrO3	1
78	1-Hydroxy-1,7-dimethyl-4-isopropyl-2,7-cyclodecadiene	35.087	Na	C15H26O	1
79	Viridiflorene	35.376	Viridiflorene	C15H24	8
80	Dodecylpalmitate	35.978	Dodecylpalmitate	C28H56O2	10
81	Sec-Butyl 2,3,4,6-tetra-methyl-.beta.,D-galactopyranoside isomer	36.292	Na	C14H28O6	2
82	1,3-EPIMANOYL OXIDE	36.366	Epimanoyl oxide	C20H34O	8
83	Pentadecane	36.435	Pentadecane	C15H32	10
84	Cyclodecane, octyl	36.599	Octylcyclodecane	C18H36	5
85	Labd-14-ene, 8,13-epoxy-, (13R)-	37.211	Na	C20H34O	10
86	5.alpha.-allyl-6.alpha.-hydroxy-5.beta.,9.beta.-dimethyl-trans-decalin-1-one	37.726	Na	C15H24O2	3
87	Naphthalene, 7-butyl-1-hexyl	38.181	Na	C20H28	10
88	1,1,7,12-tetramethyl-8-ethyl-1,2,3,4,9,10,11,12-octahydrophenanthrene	38.718	Na	C20H30	10
89	Benzamidine, 4-(4-pentylphenyl)-	39.442	Na	C18H22N2	1
90	3-Eicosene	39.652	Na	C20H40	10
91	Eicosane	40.011	Eicosane	C20H42	10
92	Sulfurous acid, hexyl nonyl ester	40.208	Na	C15H32O3S	10
93	2-Cyclohexen-1-ol, 3-methyl-6-(1-methylethyl)	40.372	Na	C10H18O	10
94	1-Acetyl-2-amino-3-cyano-7-isopropyl-4-methylazulene	40.546	Na	C17H18N2O	1
95	2-Hexadecen-1-ol, 3,7,11,15-tetramethyl	40.682	Phytol	C20H40O	10
96	1,1,8,9A-tetramethyl-2,3,5,6,7,9a-hexahydro-1h-benzo[a]cycloheptene	40.915	Na	C15H24	10
97	2-n-Heptylcyclopentanone	41.419	Na	C12H22O	10
98	7,11,15-TRIMETHYL,3-METHYLENE-1-HEXADECENE	41.645	Neophytadiene	C20H38	10
99	Stearic acid	42.294	Stearic acid	C18H36O2	10
100	Abietyl alcohol, dehydro	42.844	Abietyl alcohol, dehydro	C20H30O	2
101	Oxalic acid, hexadecyl propyl ester	43.093	Na	C21H40O4	7
102	1,4a.beta.-Dimethyl-7-isopropyl-2,3,4,4a,9,10-hexahydrophenanthrene	43.238	Na	C19H26	10
103	5-Diazo-1-(2′-methyl-4′-nitrophenylazo)-1,3-cyclopentadiene	43.79	Na	C12H9N5O2	10
104	(1.alpha.,2a.alpha.,8b.alpha.)-1,2,2a,8b-Tetrahydro-8b-hydroxy-1	44.228	Na	C17H13NO	10
105	2-Methyl-5,6-diphenyl-1,2,4-triazin-3(2H)-one	44.27	Na	C16H13N3O	1
106	Dehydroabietal	44.302	Dehydroabietal	C20H28O	3
107	8-Methoxy-4-methylbenzo[g]quinoline-5,10-dione	44.617	Na	C15H11NO3	10
108	Phenol, 4-methoxy-2-[5-(4-methylphenyl)-3-pyrazolyl)-	45.092	Na	C17H16N2O2	10
109	Pentatriacontane	45.127	Pentatriacontane	C35H72	10
110	Tetracyclo[16.1.0.0(2,9).0(10,17)]nonadeca-2(9),10(17)-diene, 19,19-dimethyl-	45.232	Na	C21H32	10
111	1,2-Dihydro-1-methyl-2-trifluoroacetylmethylenequinoline	45.506	Na	C13H10F3NO	10
112	Simvastatin	45.671	Simvastatin	C25H38O5	3
113	Methyl-9-anthracenemethanamine	45.764	Na	C16H15N	7
114	4,8,12,16-Tetramethylheptadecan-4-olide	46.318	Na	C21H40O2	2
115	2-Ethyl-3-phenyl-2-butene-1-al	47.678	Na	C12H14O	2
116	Hinokione methyl ether	47.827	Hinokione methyl ether	C21H30O2	10
117	8-Isopropyl-1,3-dimethylphenanthrene	47.873	Na	C19H20	1
118	Cyclohexanone, 2-butyl	48.334	Na	C10H18O	10
119	17-Methoxy-d-homo-18-norandrosta-4,8,13,15,17-pentaen-3-one	48.756	Na	C20H22O2	10
120	5,8,11,14-Eicosatetraynoic acid, methyl ester	48.899	Na	C21H26O2	4
121	MARGOCIN	48.964	MARGOCIN	C20H26O2	1
122	Phenanthro[3,2-b]furan-7,11-dione, 1,2,3,4-tetrahydro-4,4,8-trimethyl-	49.337	Na	C19H18O3	10
123	1,4-Bis(2-chloro-1,1-dimethylethyl)benzene	49.547	Na	C14H20Cl2	2
124	Phenol, 4-methoxy-2-[5-(4-methylphenyl)-3-pyrazolyl)-	49.67	Na	C17H16N2O2	10
125	6-Hydroxy-2,2,5,7,8-pentamethyl-4-phenyl-2H-1-benzopyran	50.552	Na	C20H22O2	10
126	Phenanthro[3,2-b]furan-7,11-dione, 1,2,3,4-tetrahydro-4,4,8-trimethyl-	51.077	Na	C19H18O3	10
127	2-Phosphabicyclo[3.1.0]hex-3-ene, 2,6,6-trimethyl-3,4-diphenyl-	51.481	Na	C20H21P	5
128	2,3-(anti)-Epoxy-1,4-dimethyl-1,4,6,13-tetrahydrobenzo[g]pyridazino[1,2-b]	51.743	Na	C18H16N2O3	1
129	Benzenamine, N,N-diethyl-4-[2-(4-nitrophenyl)ethenyl]-	52.901	Na	C18H20N2O2	10
130	2,6-Bis(1,1-dimethylethyl)-4-phenylmethylenecyclohexa-2,5-dien-1-one	53.235	Na	C21H26O	9
131	2H-Pyrazole, 3-amino-5-methyl-2-(4-nitrophenyl)-4-phenyl-	53.378	Na	C16H14N4O2	10
132	1-(2-Isopropyl-phenyl)-3,6,6-trimethyl-1,5,6,7-tetrahydro-indazol-4-one	53.687	Na	C19H24N2O	10
133	3-(N,N-Diethylamino)-5-iodoaniline	54.347	Na	C10H15IN2	10
134	17-Methoxy-d-homo-18-norandrosta-4,8,13,15,17-pentaen-3-one	54.378	Na	C20H22O2	1
135	Heneicosane, 11-(1-ethylpropyl)	56.17	Na	C26H54	3
136	2-nitro-5a,6,6-trimethyl-5a,6-dihydro-12h-indolo[2,1-b][1,3]benzoxazine	56.338	Na	C18H18N2O3	10
137	6H-Dibenzo[b,d]pyran-1-ol, 6,6,9-trimethyl-3-pentyl-	57.279	Cannabinol	C21H26O2	10
138	Sulfurous acid, pentadecyl 2-propyl ester	57.372	Na	C18H38O3S	10
139	Hexadecane, 2,6,10,14-tetramethyl	57.927	Na	C20H42	10
140	Triacontane	59.093	Triacontane	C30H62	10
141	P-Methyl-o-(phenylethynyl)phenol	81.661	Na	C15H12O	3

### Acute toxicity

No mortality and behavioral change were observed at specified doses to confirm acute toxicity of the samples. According to the assay, dose up to 2,000 mg/kg was considered as safe for essential oil of *Isodon rugosus*.

### Writhing test

A dose dependent response was observed in acetic acid induced writhing test for the assessment of analgesic activity. The mean writhes of the standard drug at 10 mg/kg, was 21.83 ± 0.60 with 70.29% inhibition. The essential oil sample exhibited mean inhibition of 31.50 ± 1.28 with 57.14% at 100 mg/kg, while, at 50 mg/kg it exhibited mean inhibition of 41.00 ± 0.57 with 44.21%. At 100 mg/kg, Ir.EO and positive control exhibited a response of 57.14 and 70.29% respectively as shown in Table 2.

**Table 2:** Percent anti-nociceptive potential of essential oil following acetic acid induced writhing model.

**Samples**	**Dose (mg/kg)**	**Mean writhes**	**% Analgesic activity**
Negative cont	–	73.50 ± 0.61	0.00
Ir.Eo	50	41.00 ± 0.57^***^	44.21
Ir.Eo	100	31.50 ± 1.28^***^	57.14
Positive cont	10	21.83 ± 0.60^***^	70.29

Ir.Eo, Essential oil isolated from Isodon rugosus; Mean writhes are represented as mean ± SEM.

*** *P < 0.001*.

### Formalin test

The results obtained from formalin test are shown in the Table 3. The formalin injection (2%, i.p) to the animals revealed a typical biphasic licking response. In the control group, duration of licking was observed as 57.33 ± 0.88 and 67.00 ± 0.93 s for early (0–5 min) and late phase (15–30 min) respectively. Pre-treatment of mice with various concentrations of essential oil (50 and 100 mg/kg) produced a significant effect on the duration of licking in both phases. A dose of 100 mg/kg of Ir.EO brought a significant reduction in paw licking of 54.36 and 43.28% in early and late phase respectively. In comparison, the standard drug morphine (5 mg/kg i.p.) demonstrated overwhelming reduction in both phases, i.e., 79.36% (early phase/neurogenic pain) and 79.59% (late phase/inflammatory pain). The morphine in combination with naloxone exhibited 04.63 and 05.22% activity in early and late phase respectively. In comparison, Ir.EO in combination with naloxone revealed 09.01% (early phase) and 07.95% (late phase) pain inhibitions. So, the naloxone reversed the antinociceptive effect of essential oil considerably at dose of 100 mg/kg in both phases as those of morphine.

**Table 3:** Effect of Essential oil of *Isodon rugosus* on formalin induced pain in mice.

**Samples**	**Dose (mg/kg)**	**Total time spent in licking**			
		**0–5 min**	**% Inhibition**	**15–30 min**	**% Inhibition**
Negative cont.	–	57.33 ± 0.88	–	67.00 ± 0.93	–
Ir.Eo	50	39.50 ± 1.11^***^	31.10	49.67 ± 1.92^***^	25.86
Ir.Eo	100	26.16 ± 0.94^***^	54.36	38.33 ± 0.71^***^	43.28
Mor	5	11.83 ± 1.24^***^	79.36	13.67 ± 0.67^***^	79.59
Mor + Nal	5 + 1	54.67 ± 1.02^ns^	04.63	63.50 ± 1.17^ns^	05.22
Ir.Eo + Nal	50 + 1	52.16 ± 0.70^**^	09.01	61.67 ± 1.14^*^	07.95

Ir.Eo, Essential oil of Isodon rugosus; Mor, Morphine; Nal, Naloxone; Total time is represented as mean ± SEM.

* *P < 0.05*,

** *P < 0.01*,

*** *P < 0.001*.

### Hot plate test

The results obtained in the hot plate assay are shown in Table 4. The Ir.EO revealed a dose dependent increase in the latency time as that of positive control. At 15 min, the mean reaction times for 50 and 100 mg/kg body weights of essential oil were observed as 06.40 ± 0.11 and 08.16 ± 0.08 min respectively. At 90 min, i.e., last interval, the mean reaction times of the same two doses were recorded as 04.40 ± 0.20 and 06.45 ± 0.07 min respectively. In comparison, the standard drug morphine exhibited reaction times of 12.41 ± 0.11 and 09.38 ± 0.08 min at initial and last interval respectively.

**Table 4:** Effect of Essential oil of *Isodon rugosus* on hot plate induced pain in mice.

**Samples**	**Dose (mg/kg)**		**Reaction time on hot plate**			
		**15 min**	**30 min**	**45 min**	**60 min**	**90 min**
Negative cont.	–	04.51 ± 0.10	02.43 ± 0.14	03.55 ± 0.07	02.11 ± 0.15	03.20 ± 0.15
Ir.Eo	50	06.40 ± 0.11	04.18 ± 0.11	05.76 ± 0.08	04.81 ± 0.14	04.40 ± 0.20
Ir.Eo	100	08.16 ± 0.08	06.81 ± 0.13	07.91 ± 0.11	06.80 ± 0.07	06.45 ± 0.07
Mor	5	12.41 ± 0.11	11.63 ± 0.06	11.36 ± 0.08	10.45 ± 0.07	09.38 ± 0.08
Mor + Nal	5 + 1	04.35 ± 0.13	02.80 ± 0.19	04.35 ± 0.07	02.78 ± 0.10	03.56 ± 0.14
Ir.Eo + Nal	50 + 1	05.28 ± 0.13	03.31 ± 0.08	04.70 ± 0.08	03.11 ± 0.15	03.70 ± 0.05

*Ir.Eo: Essential oil of Isodon rugosus; Mor: Morphine; Nal: Naloxone; Total time is represented as mean ± SEM*.

Moreover, the recorded mean reaction time for Ir.EO with naloxone (50: 1 mg/kg) at 15 min was 05.28 ± 0.13 min. Similarly, for morphine and naloxone (5: 1 mg/kg), the mean reaction time observed was 04.35 ± 0.13 min at initial 15 min. In our experiment, we found a distinct reduction in reaction time with the administration of naloxone.

### Involvement of opioid receptors

In both hot plate and formalin models, we noticed that Ir.EO revealed a similar activity as that of morphine. The potency of Ir.EO was reduced effectively by opioid antagonist naloxone. With the use of naloxone, the decreased in reaction time in hot plate method and reversing the paw licking in formalin assay confirmed the possible involvement of opioid receptors.

### Antioxidant assays

The antioxidant potential of Ir.EO using DPPH and ABTS free radicals scavenging methods are shown in Table 5.

**Table 5:** Antioxidant activity of essential oil of *Isodon rugosus* at various concentrations.

**Test sample**	**Free radicals**	**Conc. 62.5 μg/ml**	**Conc. 125 μg/ml**	**Conc. 250 μg/ml**	**Conc. 500 μg/ml**	**Conc. 1,000 μg/ml**	**IC_50_ μg/ml**
EO	DPPH	33.00 ± 1.15^***^	41.33 ± 0.88^***^	46.33 ± 0.33^***^	56.67 ± 0.67^***^	63.67 ± 1.20^***^	338
EO	ABTS	39.67 ± 1.76^***^	51.00 ± 0.57^***^	56.67 ± 1.20^***^	62.00 ± 0.57^***^	64.33 ± 0.88^***^	118
A.A	DPPH	77.33 ± 0.88	79.67 ± 0.88	83.00 ± 1.73	86.33 ± 1.45	89.00 ± 1.73	<62.5
A.A	ABTS	75.00 ± 0.57	78.00 ± 1.15	81.67 ± 0.67	85.67 ± 0.33	91.33 ± 0.88	<62.5

EO, Essential oil; A.A, Ascorbic acid.

*** *P < 0.001*.

### DPPH assay

The observed percent inhibitions for Ir.EO using DPPH free radicals was 63.67 ± 1.20, 56.67 ± 0.67, 46.33 ± 0.33, 41.33 ± 0.88, and 33.00 ± 1.15% at concentrations of 1,000, 500, 250, 125, and 62.5 μg/ml respectively. The calculated IC_50_ value from the dose response curve was 338 μg/ml. In comparison, the standard drug ascorbic acid exhibited 89.00 ± 1.73, 86.33 ± 1.45, 83.00 ± 1.73, 79.67 ± 0.88, and 77.33 ± 0.88% inhibitions at 1,000, 500, 250, 125, and 62.5 μg/ml respectively with an IC_50_ value of <0.1 μg/ml.

### ABTS assay

In ABTS assay, Ir.EO attained 64.33 ± 0.88, 62.00 ± 0.57, 56.67 ± 1.20, 51.00 ± 0.57, and 39.67 ± 1.76% inhibitions at 1,000, 500, 250, 125, and 62.5 μg/ml respectively. The calculated IC_50_ for Ir.EO in scavenging ABTS free radicals was 118 μg/ml. In this assay, ascorbic acid demonstrated 91.33 ± 0.88, 85.67 ± 0.33, 81.67 ± 0.67, 78.00 ± 1.15, and 75.00 ± 0.57% inhibitions at 1,000, 500, 250, 125, and 62.5 μg/ml respectively attaining an IC_50_ value of <0.1 μg/ml.

### Cholinesterase inhibition assay

In AChE inhibitory assay, Ir.EO exhibited concentration dependent inhibitions against the enzymes (Table 6). Ir.EO showed 67.50 ± 1.04% AChE inhibition at 1.0 mg/ml concentration with IC_50_ of 93.56 μg/ml. Similarly, the observed inhibitory potential against BChE at the same tested concentration as AChE was 61.33 ± 0.67% with an IC_50_ of 284.19 μg/ml. In comparison, the standard drug galanthamine exhibited 0.371 and 3.324 μg/ml IC_50_ against AChE and BChE respectively.

**Table 6:** Anticholinesterase activity of essential oil of *Isodon rugosus* at various concentrations.

**Test sample**	**Enzymes**	**Conc. 62.5 μg/ml**	**Conc. 125 μg/ml**	**Conc. 250 μg/ml**	**Conc. 500 μg/ml**	**Conc. 1,000 μg/ml**	**IC_50_ μg/ml**
E.Oil	AChE	42.30 ± 0.47	56.46 ± 1.27	57.00 ± 0.57	62.67 ± 0.88	67.50 ± 1.04	93.56
E.Oil	BChE	36.56 ± 0.97	41.95 ± 2.01	49.87 ± 1.67	56.00 ± 1.15	61.33 ± 0.67	284.19
Gal	AChE	74.00 ± 1.00	79.66 ± 1.85	85.00 ± 1.73	87.46 ± 1.79	94.83 ± 1.92	<62.5
Gal	BChE	63.83 ± 0.92	71.16 ± 0.92	77.83 ± 1.09	83.16 ± 1.42	88.00 ± 1.25	<62.5

*Data is expressed as Mean±SEM; Gal and E.Oil are abbreviated for Galanthamine and Essential oil respectively*.

## Discussion

In our designed work, the essential oil of *I. rugosus* was evaluated for antinociceptive, antioxidant, and anticholinestease potentials. The essential oils of plants are sources of wide variety of bioactive compounds (Dehpour et al., 2009). The pharmacological potentials of essential oil can be attributed to the hydropholic nature of its components and the same nature of our body cell membranes (Ait-Ouazzou et al., 2011). Various components of essential oils can easily get distributed to different compartments of our body including the central nervous system (Lambert et al., 2001; Vyas et al., 2008). In our current investigational study, the antinociceptive potential of essential oil was recorded with significant results. The possible mechanism of antinociceptive activity of Ir.EO was figured out as the central pathway due to involvement of opioid receptors. Recently, we have also reported the antinociceptive potential of chloroform fraction of *I. rugosus* following the same mechanism. The antinociceptive potential of essential oil may be due to the presence of large number of bioactive compounds as obvious from its GC-MS analysis. Among the identified compounds, we also observed some of the bioactive compounds previously reported with analgesic potentials. These compounds include α-copaene, germacrene D, β-caryophyllene, α-caryophyllene, aromadendrene, calamenene, viridiflorol, mansonone C, t-muurolol, α-cadinol, azunol, phytol, neophytadiene, and simvastatin. In short, α-copaene has been reported to possess strong analgesic and antioxidant potentials (Him et al., 2008; Chen et al., 2011; Costa et al., 2011). Likewise, germacrene D also possesses analgesic and antioxidant effects (Del-Vechio-Vieira et al., 2009; Victoria et al., 2012). β-Caryophyllene is also reported with its analgesic and antioxidant potentials (Calleja et al., 2013; Klauke et al., 2014). The antinociceptive activity of aromadendrene has also been demonstrated (Cruz et al., 2011). Similarly, α-caryophyllene has been reported for the treatment of body inflammatory pain (Pianowski et al., 2004). The analgesic and antioxidant effects of calamenene have also been demonstrated with significant results (Azevedo et al., 2013; Imam et al., 2014). Viridiflorol, a well-known bioactive compound is also reported to possess analgesic and radical scavenging potentials (Perry et al., 1997; do Amaral et al., 2007). Moreover, Mansonone C (including its reduced form) is also responsible for direct antioxidant activity (Villamil et al., 1990). T-muurolol has been verified for inhibitory activity against DPPH free radicals (Cheng et al., 2004). The analgesic activity of α-cadinol has also been reported with notable results (Boutaghane et al., 2011). The antinociceptive aspects of azunol has also been published previously (Ushiyama et al., 2009). In the same way, ledene has also been reported to possess analgesic activity (Alagammal et al., 2012). A well-known compound, i.e., phytol, is famous for its antioxidant potential along with its antinoceptive potential (Santos et al., 2013). Neophytadiene is also among the famous analgesic and antioxidant candidates (Jayashree et al., 2015). Similarly, simvastatin is also previously reported with its analgesic and antioxidant potentials (Carneado et al., 2002; Chen et al., 2013).

Literature review and the results of our current investigations go parallel with sound correlation. The traditional use of *I. rugosus* as analgesic is efficiently verified in the current research project, along with the identification of bioactive compounds.

Beside the antioxidant potential of Ir.EO, we also evaluated its AChE and BChE inhibitory potentials. Among other pathological targets of Alzheimer disease, inhibitions of cholinesterase and free radicals are also vital targets. Among the clinically approved anti-Alzheimer drugs, four are cholinesterase inhibitors, which signify the importance of this target in the symptomatic management of the disease. In the current study, we observed a moderate *in-vitro* cholinesterase inhibitory activity of Ir.EO. In AChE and BChE inhibitory assays, Ir.EO showed concentration dependent inhibitions against the enzymes with IC_50_ values of 93.56 and 284.19 μg/ml respectively. Though the *in-vitro* enzyme inhibitory activity of essential oil was low in comparison to galanthamine, yet, we hypothesize that it will have more availability at the target site. However, further studies are required regarding *in-vivo* efficacy of our tested essential oil.

## Conclusion

Based on the literature survey regarding the medicinal aspects of *I. rugosus* and the results of current investigational study, it may be deduced that the essential oil of *I. rugosus* is a good source of natural bioactive compounds containing numerous analgesic and antioxidant agents. Its antioxidant potentials along with cholinesterase inhibitory activity will be potentially effective in the management of Alzheimer's disease patients. It may also be inferred that further exploitation of essential oil of *I. rugosus* may lead to the development of new analgesic and/or anti-Alzheimer drug candidates.

## Author contributions

AZ and SA carried out experimental work, data collection and literature search under the supervision of AS. FU helped as co-supervision of the research work. MA, NM and UR drafted the manuscript for publication. AS supervise the overall project and make the final version of publication. All the authors have read and approved the final manuscript for publication.

### Conflict of interest statement

The authors declare that the research was conducted in the absence of any commercial or financial relationships that could be construed as a potential conflict of interest.
